# Effects of *Loofah cylindrica* extract on learning and memory ability, brain tissue morphology, and immune function of aging mice

**DOI:** 10.1515/biol-2021-0046

**Published:** 2021-04-23

**Authors:** Limin Dong, Chunjie Liu

**Affiliations:** Department of Basic Medicine, Luohe Medical College, Luohe 462000, China; Department of Pharmacy, Luohe Medical College, Luohe 462000, China

**Keywords:** *Loofah cylindrica* extract, aging, d-galactose, learning and memory ability, morphology, immune regulation

## Abstract

In this study, we aimed to observe the effects of *Loofah cylindrica* (LC) extract on learning and memory ability, brain tissue morphology, and immune function of aging mice. Kunming mice were selected and randomly divided into a control group, a positive control group, an aging group, and three dose groups. Three dose groups were administered 187.5, 375, and 750 mg/kg of LC extract, respectively. Except for the control group, the mice in any other group were continuously subcutaneously injected d-galactose on the back and neck. Platform tests and Morris water maze (MWM) were adopted to test the learning and memory ability of each group. The brain index, thymus index, and spleen index of each group were determined by the organ-to-body ratio method. The enzyme-linked immunosorbent assay was performed to measure the concentration of cytokines interleukin 2 and interferon gamma and the proliferation activity of T lymphocytes in mouse serum. In addition, the hematoxylin and eosin staining was employed to observe the morphological changes in mouse brain tissues of each group. The results show that the aging group made more errors in the platform test, had longer escape latency, shorter swimming time in the platform quadrant, and fewer platform crossings in the MWM; much fewer brain tissue cells; and smaller brain index, thymus index, and spleen index. The LC extracts (375 and 750 mg/kg) can significantly antagonize the changes in the above indices. It can be concluded that LC extract can improve the learning and memory of aging mice, enhance their immune activity, and delay the aging process.

## 1 Introduction

As an inevitable stage of life, aging is mainly manifested as the weakening or loss of the body’s ability to adapt to the environment and the functional decline in various organs and tissues. Understanding and delaying aging has been a research hot spot around the world [1]. Brain aging is the most apparent manifestation of human aging. Brain aging is a very complex process, leading to changes in brain structure and function, which is caused by a variety of factors, among which the decrease in learning and memory ability is one of the most important manifestations [2,3,4]. Excessive intake of d-galactose will cause metabolic disorders and produce a large number of superoxide anion free radicals, resulting in oxidative damage to a variety of biological macromolecules, affecting gene expression and regulation system, leading to decreased cellular transcription level, and causing aging changes [5,6,7]. Literature has reported that the application of d-galactose to establish an animal aging model can widely be used in the study of aging mechanisms and the screening of antiaging drugs [8,9,10]. *Loofah cylindrica* (LC) is a cucurbitaceous plant with rich nutritional value. The main nutrients include protein, fat, vitamins, sugars, alkaloids, saponins, as well as glutamic acids. Traditional Chinese medicine holds that LC can clear heat, cool blood, cure rheumatism, reduce phlegm, dredge the meridians, promote blood circulation, and kill insects [11]. LC extract has been reported to have functions like antistress, antiviral, growth promotion, and immune enhancement [12]. Research results have shown that the extracts from LC fruit, leaf, and vine can promote acid phosphatase activity and macrophage phagocytosis in peritoneal macrophages of normal mice [13], thereby affecting the immune functions of normal mice.

The flavonoids separated from LC may be the reason for the antioxidant effects of LC extract in the body [14]. However, there is not much known about the immunomodulatory effect of LC extracts on aging mice. Despite its wide use in antiaging cosmetics, few scholars have explored the delaying effect of LC extract on brain aging.

As *Loofah* is a medicine and food plant, it has been used as an adjuvant treatment in a variety of diseases, with only minor side effects. The safety of *Loofah* root extract has been reported in the literature. When the maximum tolerated amount of *Loofah* root extract (110 g crude drug/kg) was given to mice by gavage, the animals had no symptoms of systemic poisoning and died [15]. The toxicological test of *Loofah* extract showed that its oral decoction was 100 times higher than the clinical dose for 10 days. Morphological detection of 11 organs such as the brain, heart, and liver showed no obvious damage, indicating low toxicity and safe use [16]. Based on the previous research, this study used d-galactose continuous administration to create the aging model of mice and studied the effects of *Loofah* extract on behavioral memory and immune regulation of mice so as to provide the basis for the development of new drugs and clinical application.

## 2 Methodology

This experiment is conducted through the design platform and water maze experiment on mice behavior. Test memory ability in mice and the little mouse and thymus and spleen index can be determined through detection of serum cytokine interleukin 2 (IL-2). The concentration of interferon gamma (IFN-γ) was expected to detect drug immunity ability. The histomorphology and design experiment are expected to allow observation of brain neurons’ changes from validation drugs for brain protection.

### 2.1 Test drugs

The *Loofah* extract is a proportional extract. After the *Loofah* fruit dries, 70% ethanol reflux is used three times, and the extract is concentrated (1:1). The petroleum ether is extracted, the lower extraction liquid is concentrated, and vacuum drying is performed to obtain the extract powder. Extract powder 100%, put through 80 mesh sieve and obtained in form of brown fine powder, provided by Xi’an Sen Ran Biotechnology Co., Ltd. Naofukang (Piracetam tablet) was purchased from Shanghai Xinyi Pharmaceutical Co., Ltd. (batch number: 20130903).

### 2.2 Animals

Seventy-two SPF grade healthy male Kunming mice were selected, each of which weighted between 25 and 35 g. The mice were provided by Henan Experimental Animal Center (license number: SCXK (pre) 2010-0002).


**Ethical approval:** The research related to animal use has been complied with all the relevant national regulations and institutional policies for the care and use of animals and has been approved by the Medical Ethics Committee of Luohe Medical College (Luohe, China).

### 2.3 Reagents

d-Galactose (Shanghai Reagent No.2 Factory); RPMI-1640 culture medium (Shanghai Yuanlong Biotechnology Co., Ltd); concanavalin (ConA), methyl thiazolyl tetrazolium, dimethyl sulfoxide (Sigma); IFN and IL-2 enzyme-linked immunosorbent assay (ELISA) kit (eBioscience). The remaining chemical reagents were produced domestically and confirmed analytically pure.

### 2.4 Instruments

DT-200 mouse platform (Chengdu Taimeng Technology Co., Ltd), Morris water maze (MWM) (Chengdu Taimeng Technology Co., Ltd), high-speed refrigerated centrifuge (Shanghai Canspec Scientific Instruments Co., Ltd), ELX-800 microplate reader (American Bert Co., Ltd), UV-Vis spectrophotometer (Shanghai Precision Scientific Instrument Co., Ltd), and CO_2_ incubator (BPN-150CH).

### 2.5 Method

The mice were randomly divided by weight into a control group, Naofukang group, an aging group, and three doses groups. The *Loofah* extract dose group 1 was 750 mg/kg, dose group 2 was 375 mg/kg, and dose group 3 was 187.5 mg/kg [17,18]. Except for the control group, the mice were continuously and subcutaneously injected 150 mg/kg d-galactose on the back and neck for 8 consecutive weeks to induce the aging model. Meanwhile, the mice in the positive control group were intragastrically administered 800 mg/kg Naofukang. The control group and aging group were intragastrically administered 0.9% of normal saline. After 8 weeks, the learning and memory ability of each group was tested.

#### 2.5.1 Platform test

The mice were left to adapt to the environment for 3 min. Then the bottom of the box was energized with 36 V alternating current. The usual response is to jump to the platform to avoid electric shock. Then each mouse was placed on the insulating platform. The prelearning latency was defined as the duration between the jumping onto the platform and the first jumping off the platform. The number of errors was defined as the number of electric shocks in 5 min [19]. The latency and number of errors were taken as the learning score of each mouse. After 24 h, the same operations were repeated once, and the postlearning latency and number of errors were recorded as the memory score of each mouse.

#### 2.5.2 MWM positioning navigation test and space exploration test

The MWM was divided into four quadrants, namely, I, II, III, and IV. A video camera connected to the display system was placed above the maze to record the movement of each mouse simultaneously. During each test, the reference objects around the maze remained unchanged. The positioning navigation test was carried out in the first 5 days, and the space exploration test was conducted on the sixth day.

##### 2.5.2.1 Positioning navigation test

This test was carried out once a day in the first 5 days. During each test, a mouse was put into the water in each quadrant with head facing the pool wall. The automatic camera system plus the computer analysis and processing system recorded the escape incubation period – the time of finding the platform within 120 s. If the mouse failed to find the platform within 120 s, it would be guided to find the platform and remain there for 15 s. The escape latency in this case was recorded as 120 s.

##### 2.5.2.2 Space exploration test

On the sixth day, the platform was removed. During the test, a mouse was put into the water in a random quadrant with head facing the pool wall and fixed in that quadrant afterward. The swimming time in the platform quadrant and the number of platform crossings in 120 s were recorded.

#### 2.5.3 Determination of brain, thymus, and spleen indexes

After the learning and memory tests, the mice were accurately weighed and killed by removing the cervical spine. The whole brain, thymus, and spleen tissues were aseptically removed on an ultraclean workbench, repeatedly washed with precooled saline, and dried with filter paper. Then the tissues were weighed on an electronic balance. Finally, the index of each organ was calculated:

\text{Organ}\hspace{.5em}\text{index}=\text{organ}\hspace{.5em}\text{mass}\hspace{.5em}\text{(mg)}/\text{mouse}\hspace{.5em}\text{weight}\hspace{.5em}\text{(g)}]

#### 2.5.4 T-lymphocyte transformation test

After the mice were killed, the spleen was taken by conventional aseptic operations and prepared for a spleen cell suspension. The cell concentration was adjusted to 1 × 10^6^/mL. On a 96-well cell culture plate, 0.2 mL spleen cells and 15 μL ConA were added to each well, and the final concentration was adjusted to 6 μg/mL. Three replicate wells were arranged for each sample, and a well without ConA was taken as the control. Then the culture plate was covered and placed for 72 h in an incubator with 5% CO_2_ and 37°C. After that the A value was measured at the wavelength of 560 nm in the microplate reader (American Bert Co., Ltd). The stimulus index (SI) was calculated by dividing the absorbance value of test wells by that of the control well.

#### 2.5.5 ELISA of the concentration of cytokines IL-2 and IFN-γ in serum

The whole blood sample was centrifuged for 20 min. Then the supernatant was taken for detection. The concentration of cytokine IL-2 and IFN-γ in the serum was determined by the detection method specified by the ELISA kit provided by eBiosicence. The optical density (OD) of each well was measured at the wavelength of 450 nm in turn with the microplate reader.

#### 2.5.6 Morphological observation

After the platform tests, each group of mice was killed immediately. The brain tissue was quickly taken, fixed in neutral formalin solution, embedded in paraffin, sectioned, and hematoxylin and eosin stained. Then morphological changes in brain tissue neurons were observed under an optical microscope.

### 2.6 Statistical methods

All data were analyzed using SPSS21.0 statistical software, and measurement data were expressed as mean ± SD (\overline{\text{X}}\pm \text{S}]). One-way ANOVA was used for comparison between groups, and least significant difference method was used for comparison between groups. *P* < 0.05 was considered statistically significant.

## 3 Results

### 3.1 Effects on learning and memory in d-galactose-induced aging mice

**Figure 1:**
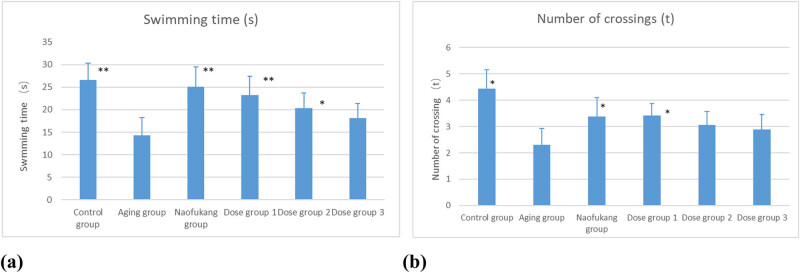
Effect of LC extract on latent period and error number in aging mice induced by d-galactose compared with blank group (\overline{\text{X}}\pm \text{S}], *n* = 10). (a) Latent period and (b) number of errors. *P < 0.05, and **P < 0.01.

During the learning ability test, the mice in the aging group had a much longer latency of jumping up and made significantly (*P* < 0.01) more errors than those in the control group. The mice in the Naofukang group and the dose group of 375 mg/kg had a much shorter latency and made fewer errors than those in the aging group. The differences are also statistically significant (*P* < 0.01; Figure 1a).

In the memory ability test, the mice in the aging group had a much shorter latency of jumping off and made many more errors than those in the control group. The differences are statistically significant (*P* < 0.01). The mice in the Naofukang group and the dose groups of 375 and 750 mg/kg had a much longer latency and made fewer errors than those in the aging group. The differences were statistically significant (*P* < 0.01; Figure 1b).

### 3.2 Effects on MWM positioning navigation and space exploration

In the positioning navigation test, the escape latency of each group gradually shortened with the extension of training time. From the second day, the escape latency of aging group was much longer than that of the control group (*P* < 0.01), suggesting an obvious damage in learning and memory function.

**Figure 2:**
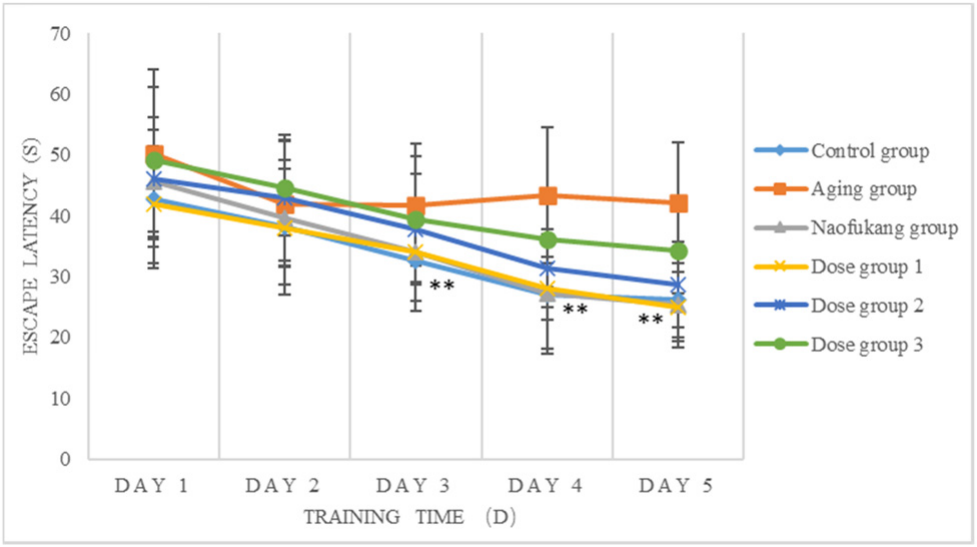
The effects of LC extract on the escape latency in MWM positioning navigation (\overline{\text{X}}\pm \text{S}], *n* = 10). Note: compared to the aging group *P < 0.05 and **P < 0.01.

At the beginning of the third day of the experiment, compared with the aging group, the incubation period of finding the platform in dementia mice was significantly shortened in the *Loofah* extract 750 mg/kg group and the cerebral rehabilitation group (*P* < 0.01). There was no significant difference in the escape incubation period of the *Loofah* extract 750 mg/kg group and the cerebral rehabilitation group (*P* > 0.05; Figure 2).

**Figure 3:**
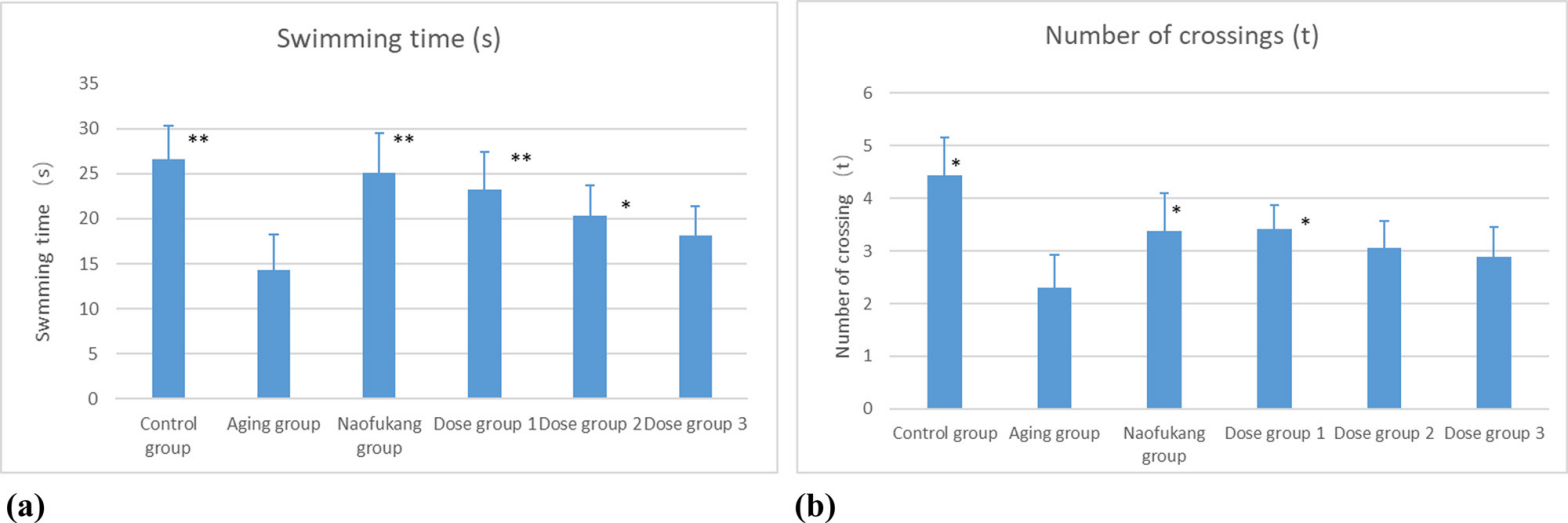
The effects of LC extract on the swimming time and number of crossings in MWM space exploration compared with blank group (\overline{\text{X}}\pm \text{S}], *n* = 10). (a) The swimming time and (b) number of crossings. *P < 0.05 and **P < 0.01.

The aging group had a much shorter swimming time in the platform quadrant (*P* < 0.01) than the control group and made fewer crossings of the platform (*P* < 0.05). The dose groups 375 and 750 mg/kg, as well as the Naofukang group, clearly extended the swimming time in the platform quadrant compared to the aging group (*P* < 0.01; Figure 3a). All dose groups increased the number of the platform crossings from the aging group’s level to different degrees. Note that dose group 750 mg/kg and Naofukang group had a significant difference in the number of crossings with the aging group (*P* < 0.05; Figure 3b).

### 3.3 Effects on brain, thymus, and spleen indices in d-galactose-induced aging mice

**Figure 4:**
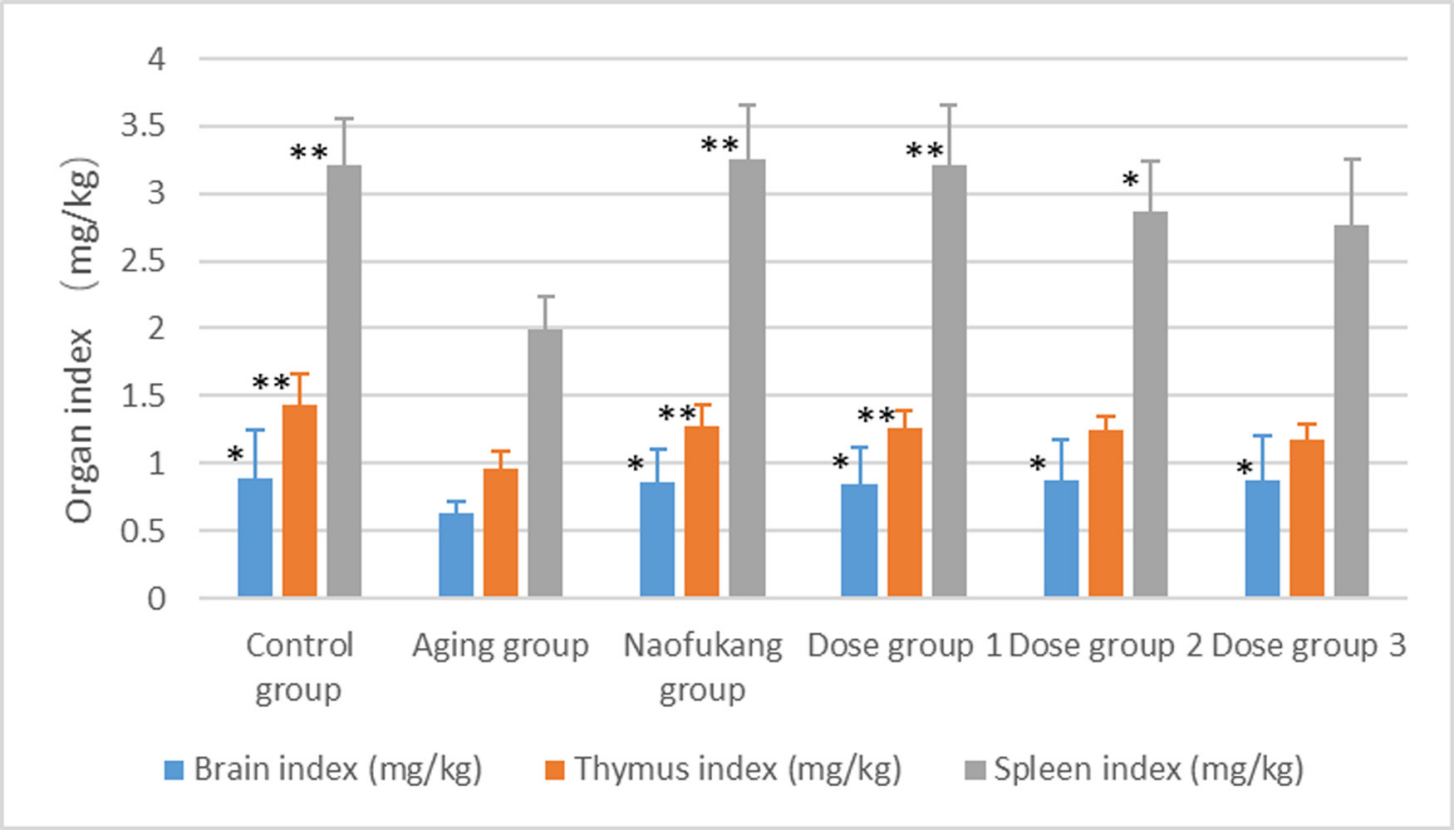
The effects of LC extract on brain, thymus, and spleen indices in d-galactose-induced aging mice (\overline{\text{X}}\pm \text{S}], *n* = 10). Note: compared to the aging group *P < 0.05 and **P < 0.01.

The aging group had a much smaller brain, thymus, and spleen indices than the control group (*P* < 0.05 to *P* < 0.01). These indices of the Naofukang group and the dose groups 375 and 750 mg/kg were much larger than those of the aging group (*P* < 0.05 or *P* < 0.01). There is no significant dose dependence (Figure 4).

### 3.4 Effects on splenic T-lymphocyte transformation function and serum cytokine IL-2 and IFN-γ in d-galactose-induced aging mice

**Figure 5:**
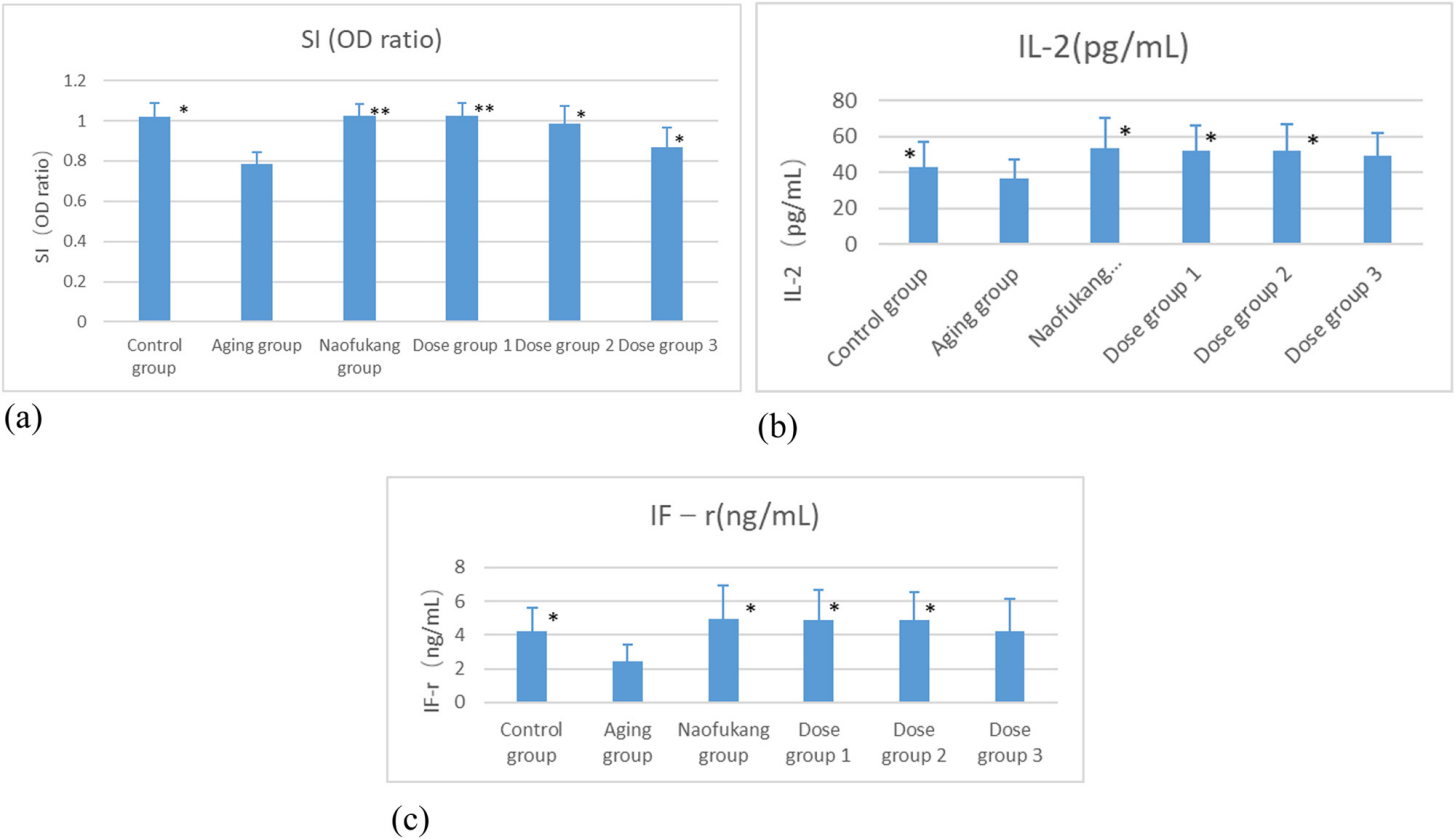
The effects of LC on immune function of aging mice and blank group were compared (\overline{\text{X}}\pm \text{S}], *n* = 10). (a) Effect on splenic T lymphocyte transformation function, (b) effect on serum cytokine IL-2, and (c) effect on IFN-γ. *P < 0.05 and **P < 0.01.

The SI of splenic T-lymphocyte transformation of the aging group was significantly lower than that of the control group (*P* < 0.05). The SI value of the Naofukang group and any dose group was significantly higher than that of the aging group (*P* < 0.05 or *P* < 0.01; Figure 5a).

The serum cytokines IL-2 and IFN-γ in the aging group were significantly smaller than those of the control group (*P* < 0.05). In addition, dose groups 375 and 750 mg/kg had significantly higher IL-2 and IFN-γ than those in the aging group (*P* < 0.05; Figure 5b and c).

### 3.5 Morphological observation of mouse brain tissue

**Figure 6:**
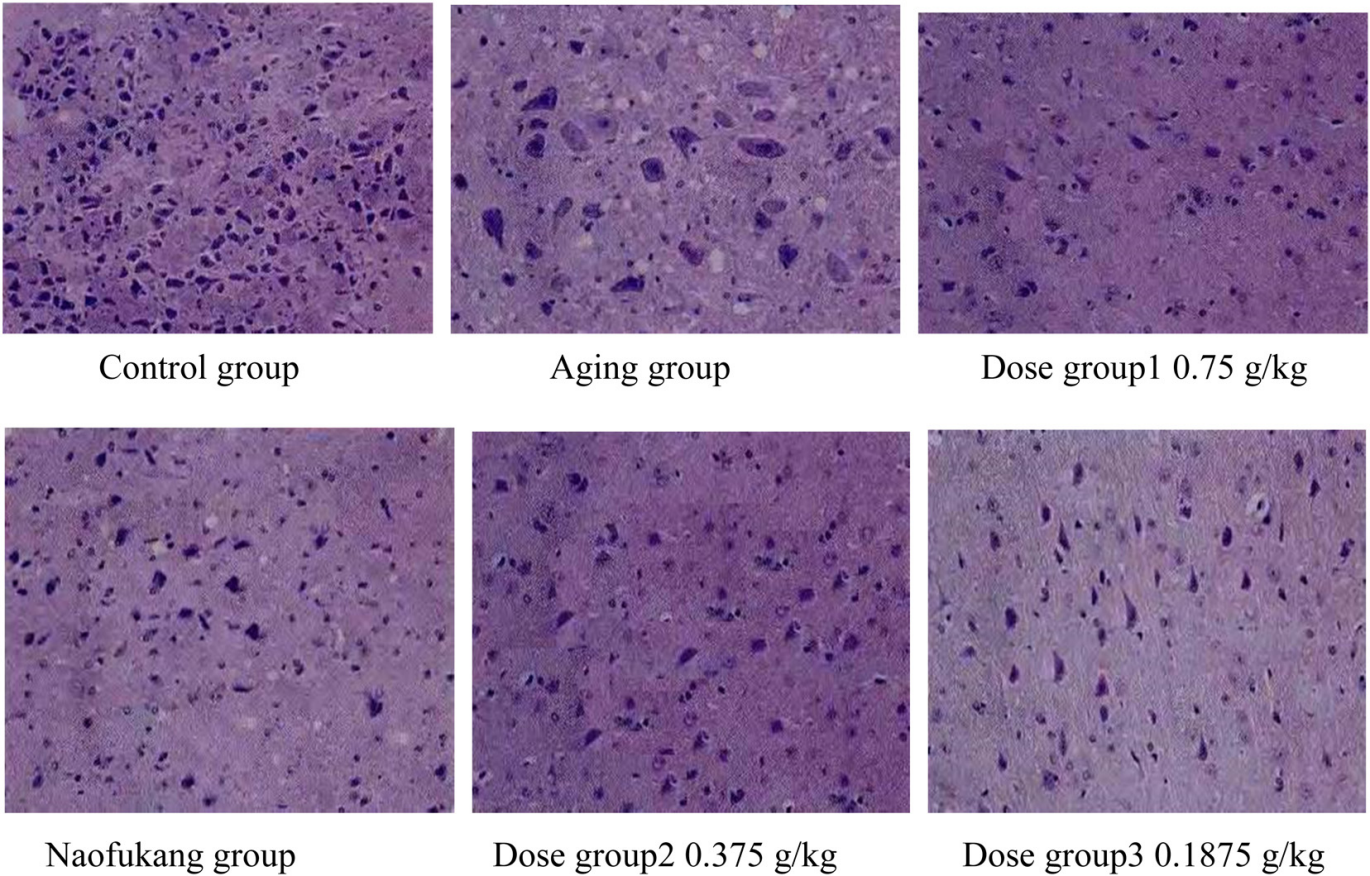
Brain cell morphology in each group.

The brain tissue cells of the control group had regular morphology and clear structure. The numerous cells were arranged neatly, without any degenerated cells. In the aging group, the cerebral cortex of the mice was thinned, the number of brain cells was significantly reduced, and the cell arrangement was disordered. Moreover, the coloration was uneven, and some nuclei were contracted. The changing morphology left some cavities. Compared with the aging group, the Naofukang group and dose groups 375 and 750 mg/kg had complete and neatly arranged cell morphology and structure, despite mild cell swelling; the number of cells was increased. As for the dose group 187.5 mg/kg, a few cavities were observed. Overall, the three doses groups all witnessed improvement in the number and arrangement of brain cells (Figure 6).

## 4 Discussion

There are many animal models for studying aging and dementia. Injecting d-galactose into mice is a common way to simulate aging animal models. This approach has been widely adopted in the pharmacological research of neurodegenerative diseases, namely, Alzheimer’s disease [20,21,22,23]. The MWM positioning navigation test reflects the spatial cognitive ability of mice, while the space exploration test reflects their spatial memory ability. The test results show that LC extract can significantly shorten the escape latency in positioning navigation test and greatly increase the swimming time in platform quadrant and the number of platform crossings in space exploration test.

The behaviors of the mice in the platform test belong to passive avoidance reflex. Thus, the test results demonstrate the effect of drugs on the whole process of memory, the escape latency, and the safety plateau period. This eliminates the interference of nonspecific effects of drugs [24]. Our test results show that the pre- and postlearning latencies of mice reduced, and the number of mistakes increased. The LC extract at any dose could extend the two latencies, lower the number of mistakes, and improve the learning and memory ability. That is why the dose groups achieved much better scores than the aging group. The results prove LC extract can improve memory from the behavioral perspective.

Microscope observation of brain tissue morphology shows that the number of cortex cells in three doses groups was much higher than that in the aging group, and the cell structure greatly improved. This means the LC extract can reduce senescence or apoptosis of brain cells by maintaining the cell morphology and structure, thereby protecting brain function and slowing down aging.

The immunological theory of aging believes that aging changes like the aging of immune organs, the changes of immune cells and cytokines, and the weakening of immune function lead to the body’s aging and mortality [25,26]. The weight changes in tissues and organs, especially vital organs, such as the brain, thymus, spleen, liver, and kidney, directly reflect the degree of aging of the body [27,28]. Among them, the thymus is the place where T lymphocytes differentiate and mature. The spleen is involved in various immune functions in the body.

In the body of the elderly, the reduction in immune function is closely associated with the variation in T cells. The cellular immune function state of the body can be judged by the lymphocyte transformation rate. IL-2 and IFN-γ, both of which are cytokines mainly produced by activated T lymphocytes, have a powerful immune regulation effect. IL-2 can promote the proliferation and differentiation of T cells, enhance the activity of T and NK cells, and induce interferon production. Meanwhile, IFN-γ can enhance the interaction between antigen-presenting cells and T cells, thereby stimulating the production of T-cell helper antibodies [29]. In addition, IFN-γ can also activate macrophages, bolstering their ability to kill pathogens and tumors and activate NK cells to improve their killing ability. The levels of IL-2 and IFN-γ are important signs of cellular immunity.

Our test results indicate that the mice of the aging group had abnormal immune indices. Their immune organs withered and lost function, the ability of lymphocyte transformation declined, and the IL-2 content in peripheral blood plunged. These phenomena are the exact pathophysiological features of aging. From the test results, it was observed that LC extract could increase the SI of splenic lymphocyte transformation of aged mice, elevate the concentration of IL-2 and IFN-γ in the serum, and significantly enhance the cellular immune function.

## 5 Conclusion

In summary, LC extract can improve the memory function of aged mice and protect the structure and function of their cells. The extract can also protect immune organs, increase IL-2 and IFN-γ levels in the serum, and boost the proliferation activity of T lymphocytes. The aging can be delayed by LC extract by enhancing body fluids and cellular immune function.
